# Incidence trends of early-onset breast cancer by lifestyle risk factors

**DOI:** 10.1186/s12885-025-13730-y

**Published:** 2025-02-21

**Authors:** Jasmiina Rantala, Karri Seppä, Johan Eriksson, Sirpa Heinävaara, Tommi Härkänen, Pekka Jousilahti, Paul Knekt, Satu Männistö, Ossi Rahkonen, Nea Malila, Eetu Mäkinen, Heidi Ryynänen, Maarit Laaksonen, Sanna Heikkinen, Janne Pitkäniemi, METCA Study Group

**Affiliations:** 1 Finnish Cancer Registry, Institute for Statistical and Epidemiological Cancer Research, Helsinki, Finland; 2 Department of Public Health, University of Helsinki, Helsinki, Finland; 3 Department of Public Health, Finnish Institute for Health and Welfare (THL), Helsinki, Finland; 4 Department of General Practice and Primary Health Care, University of Helsinki and Helsinki University Hospital, Helsinki, Finland; 5 Folkhälsan Research Center, Helsinki, Finland; 6 Singapore Institute for Clinical Science, Agency for Science, Technology, and Research, Singapore, Singapore; 7 Department of Obstetrics and Gynaecology and Human Potential Translational Research Programme, Yong Loo Lin School of Medicine, National University of Singapore, Singapore, Singapore; 8 School of Public Health, University of Sydney, Sydney, Australia; 9 Faculty of Social Sciences, Health Sciences Unit, Tampere University, Tampere, Finland

**Keywords:** Early-onset breast cancer, Risk factors, Prevention, Incidence trends

## Abstract

**Background:**

The incidence of early-onset breast cancer (< 50 years) has been increasing over the past decades with the role of modifiable, lifestyle-related risk factors remaining mostly unidentified.

**Methods:**

To investigate the role of key lifestyle factors in the incidence trends of early-onset breast cancer (EOBC), we pooled data from six health studies in Finland in 1972–2015 and combined them with breast cancer data from the Finnish Cancer Registry. The cohort consisted of 56,253 women with 397 EOBCs. The effects of risk factors (hazard ratios) on EOBC and average annual percent change (AAPC) of incidence were estimated using Poisson regression models.

**Results:**

The highest annual increase in age-standardized incidence was observed among women aged 40–49 who were overweight (AAPC 4.0%, 95% CI 0.5–7.7%), currently smoking (AAPC 3.3%, 95% CI 0.1–6.6%) or moderately physically active (AAPC 2.9%, 95% CI 0.3–5.6%).

**Conclusions:**

The increase in early-onset breast cancer incidence was highest among women aged 40–49 who were overweight, currently smoking, or moderately physically active, while no change by risk factors was found in women under 40 years of age. Our findings suggest a different type of cancer process in young adults and highlight the importance of lifestyle risk factors in the incidence of EOBC.

**Supplementary Information:**

The online version contains supplementary material available at 10.1186/s12885-025-13730-y.

## Introduction

Female breast cancer is the most commonly diagnosed cancer worldwide with an estimated 2.3 million new cases (11.7% of all cancer cases) diagnosed in 2020 [1]. It was also the leading cause of cancer death in women, accounting for 1 in 6 of them [1]. Although breast cancer is more frequently diagnosed in older women, it also represents an important disease entity in young adults [2]. In 2020, almost 670,000 new breast cancers occurred in women under the age of 50, accounting for 30% of all new breast cancer diagnoses [3]. In the Nordic countries, the incidence of early-onset breast cancer (EOBC) has been on a steady rise with an estimated 1.06% annual increase over the last 60 years [4]. The incidence rates of breast cancer at any age seem to increase along with the Human Development Index (HDI) [1], likely reflecting the differences in hormonal and lifestyle risk factors and availability of mammographies [1].

It is known that the effects of breast cancer risk factors differ in relation to age at diagnosis [5], suggesting that EOBC should be examined as its own entity. Concerning lifestyle factors, consumption of alcoholic beverages seems to increase the risk of EOBC [5], while evidence regarding the effect of tobacco smoking [6–8] remains inconclusive. Results of studies concerning the association between physical activity and risk of EOBC have been inconsistent and not fully comparable due to heterogeneity in study methods [9–11]. However, recent meta-analyses suggest that vigorous physical activity, meaning high-intensity activities such as running, jogging or swimming, may be inversely associated with the risk of EOBC [10, 11]. Additionally, a high level of education might be related to an increased risk of EOBC [12, 13], which is explained by a higher prevalence of certain breast cancer risk factors, such as high age at first birth and nulliparity, in more educated women [14].

The effect of body fatness on the risk of breast cancer varies depending on the timing of the exposure (childhood, adolescence, or adulthood) and the age at breast cancer diagnosis [5]. Being overweight as a child or a young adult seems to decrease the risk of both pre- and postmenopausal breast cancer [15, 16]. Low body adiposity seems to act inversely: low body mass at a young age predisposes to EOBC [16]. While obesity at young ages acts as a protective factor for breast cancer throughout life [15, 16], the role of adulthood obesity is more complex. Adulthood obesity decreases the risk of premenopausal breast cancer [17, 18] but increases the risk of postmenopausal breast cancer [17, 19].

Various endogenous [20] and exogenous [21, 22] hormonal factors are plausibly linked to the risk of breast cancer. The risk seems to be influenced by the duration of lifetime exposure to estrogen [23, 24] as early age at menarche [23, 25] and late age at menopause [23, 25] are positively associated with the risk of breast cancer. Likewise, a moderately increased breast cancer risk has been documented in users of hormonal contraceptives [21, 26]. However, the interaction between hormone exposure and the development of breast cancer is not straightforward as it seems to be further modified by the type and timing of the exposure. For instance, parity and low age at first childbirth have inverse effects on the risk of premenopausal breast cancer despite pregnancy causing a transient exposure to high hormone concentrations [12].

The cancer burden among young adults has been in continuous increase over the past decades, referred to as the ‘early-onset cancer epidemic’ [27]. In order to understand the role of modifiable risk factor exposure in the etiology of the rising cancer epidemic, there is a need to inspect the time trends of possible risk factors since the mid-20th century [27]. The current study aims to evaluate the role of key modifiable factors in the incidence trends of early-onset (< 50 years) breast cancer over the period 1972–2015.

## Materials and methods

### Study population

To investigate the role of key modifiable factors in the risk and incidence trends of EOBC, we used data from the Prospective METa Cohort Study of Cancer Burden in Finland (METCA) project. The METCA project comprises of pooled data from seven cohorts in Finland: The Finnish Mobile Clinic Health Examination Follow-up Survey (FMCF) [28], National FINRISK Study [29], Mini-Finland Health Survey (MFS) [28], The Alpha-Tocopherol, Beta-Carotene Cancer Prevention Study (ATBC) [30], Health 2000 Survey (H2000) [31], Helsinki Birth Cohort Study (HBCS) [32], and Regional Health and Well-being Study (ATH) [33]. The ATH study comprised two separate datasets, of which the first was conducted 2010–2011 (ATH1) and the second in 2012–2015 (ATH2) [33]. We additionally included the Helsinki Health Study (HHS) [34] cohort in the present study.

All cohorts included in this study were required to meet the following criteria: [1] includes information on potential breast cancer risk factors; [2] allows data linkage using the national personal identity code (PIC); [3] includes females aged 18–49 years. We excluded the ATBC (all subjects were males) and the HBCS (all subjects were older than 50 years) cohorts as they did not meet all the criteria, leaving us with pooled data from six cohorts.

In all cohorts, the study subjects were drawn from the Finnish general population by using some variant of random selection. The studies were conducted during the years 1972–2015. The follow-up started at the beginning of each survey and was terminated either on the date of breast cancer diagnosis, death, age 50 years, or end of follow-up (either on 31st December 2013 or 2015, depending on the cohort), whichever occurred first.

Data on smoking, alcohol consumption, body mass index (BMI), physical activity, education, parity, and use of hormonal contraceptives were obtained from the cohorts. Information on the exposures was based on either surveys or clinical examinations, in which the participation rates ranged from 51 to 94% [35]. Data obtained from the cohorts were linked to the breast cancer data from the Finnish Cancer Registry by using the personal identity code of each individual. Information on vital status was obtained from the Digital and Population Data Services Agency.

The selection of risk factors was based on previous evidence of their relationship with breast cancer and their comprehensive availability in the data. Risk factors were categorized into two or three levels of exposure as follows: smoking (never, former, current), alcohol consumption (no, moderate, heavy), BMI (< 25 kg/m^2^, 25–29.9 kg/m^2^ (overweight) and ≥ 30 kg/m^2^ (obese)), parity (yes, no), hormonal contraception use (never use, ever use), physical activity (vigorous, moderately active, inactive), education (low, intermediate, high).

Moderate alcohol use was defined as consuming < 7 drinks per week and heavy as ≥ 7 drinks per week. Concerning physical activity, inactive subjects were defined as reporting not having any physically burdening leisure time activities, moderate physical activity included light activities such as brisk walking, riding a bike, or light household chores. Subjects were classified as vigorously physically active if they exercised to maintain their physical condition, e.g. by jogging, swimming, running, playing ball games, or by doing heavy gardening or other similar activities. Subjects participating in competitive sports were also included in this category. The categorization of education level was based on birth year -specific tertiles of the years of education or ordered categories of education as follows: low education was defined as ≤ 9 years of education or reporting comprehensive school as highest education, intermediate as > 9 but < 12 years of education or reporting high school or vocational training as highest education and high education as 12 years or reporting college or university as highest education. Hormonal contraceptives were further split into the intrauterine device (IUD) and all other forms (oral, an implant, vaginal ring). The latter category consisted mainly of women using either combined estrogen-progesterone or only progesterone-containing pills. Subjects with missing information on the risk factors were categorized into their own group. The harmonization of risk factors has been described in detail previously [35].

The study was approved by The Finnish Institute for Health and Welfare (THL/679/6.02.00/2018).

### Statistical methods

We calculated age-standardized incidence rates of EO-BC during 1972–2015 and separately for five calendar periods (1972–1981, 1982–1991, 1992–2001, 2002–2011, and 2012–2015) by using the World Standard Population, and we reported them per 100 000 person-years. The overall numbers of person-years by age (six groups: 18–24, 25–29, 30–34, 35–39, 40–44, and 45–49 years) were used as weights in the standardization. We modelled the associations of calendar periods and the associations of risk factors using Poisson regression, where the cause-specific hazard of breast cancer is the product of the baseline hazard and the corresponding hazard ratios (HRs). Baseline hazard was stratified by calendar period and age at follow up (the same periods and age groups as mentioned above). In addition, we estimated the average annual percent change (AAPC) by including the calendar year as a continuous covariate in the Poisson model instead of the period and fitting the model separately in each category of the risk factors. Both the fully adjusted and unadjusted effects of risk factors and the AAPC were estimated. We have two types of missing data: individual missing values (respondent not responded to the question) and structural missing values (question not asked in the particular survey). As we could not obtain information of alcohol consumption from FINRISK in 1972, 1977, and 1982, physical activity from FMCF and MFS cohorts, parity from ATH cohorts and use of hormonal contraception from FMCF, MFS, and HHS cohorts, we treated data from these cohorts as a separate missing category in the risk factor analysis. We used R software, version 4.2.2 (R Foundation for Statistical Computing, Vienna, Austria) packages Epi, version 2.47 and popEpi, version 0.4.10 for data analysis.

## Results

A total of 397 early-onset primary breast cancers occurred among 56,253 women during 1972–2015 in the pooled cohort. Out of all the breast cancer cases, 53 were diagnosed in women aged 40 years or younger. Table 1 shows the number and proportion of subjects by risk factors and cohorts stratified by age group and calendar periods in the study data with structural and individual missing values presented separately. Cancer cases in each period and cohort are shown in Supplementary Table 1. More than one fourth (25.9%) of the subjects were overweight and 12.3% were obese. The majority of subjects reported moderate use of alcohol (55.2%), while 10.1% were heavy users. A fourth (25.2%) of the subjects were highly educated, and 41.3% had a low level of education. In age groups 18–39 and 40–49 years the prevalence of alcohol use, vigorous physical activity, nulliparity, and obesity increased during 1972–2015. In contrast, the proportion of women with low education decreased. Among women aged 40–49, an increase in the prevalence of current and former smoking and moderate physical activity was observed, while the prevalence of overweight decreased.

**Table 1:** Baseline characteristics of study subjects in the METCA data with individual missing (missing) and structural missing (NA) values

Age group		18–39					40–49 18–49					
**Period**		1972–1981	1982–1991	1992–2001	2002–2011	2012–2015	1972–1981	1982–1991	1992–2001	2002–2011	2012–2015	All
**Number of subjects by entry to cohort**		9760	3025	4541	4629	12,008	6055	1772	4048	3604	6811	56,253
**Risk factor and cohort**												
**Smoking**	No	5957 61.0%	1281 42.3%	2067 45.5%	1932 41.7%	5570 46.4%	4707 77.7%	1162 65.6%	1917 47.4%	1552 43.1%	3386 49.7%	29,531 52.5%
	Ex-smoker	1100 11.3%	801 26.5%	894 19.7%	1066 23.0%	3176 26.4%	378 6.2%	253 14.3%	908 22.4%	941 26.1%	1933 28.4%	11,450 20.4%
	Current smoker	2673 27.4%	934 30.9%	1553 34.2%	1601 34.6%	2956 24.6%	932 15.4%	344 19.4%	1150 28.4%	1049 29.1%	1350 19.8%	14,542 25.9%
	Missing	30 0.3%	9 0.3%	27 0.6%	30 0.6%	306 2.5%	38 0.6%	13 0.7%	73 1.8%	62 1.7%	142 2.1%	730 1.3%
**Alcohol consumption**	No	1912 19.6%	99 3.3%	283 6.2%	366 7.9%	1363 11.4%	1467 24.2%	140 7.9%	294 7.3%	280 7.8%	755 11.1%	6959 12.4%
	Moderate	2663 27.3%	1064 35.2%	3245 71.5%	3500 75.6%	8580 71.5%	1048 17.3%	557 31.4%	2996 74.0%	2644 73.4%	4744 69.7%	31,041 55.2%
	Heavy	280 2.9%	90 3.0%	453 10.0%	737 15.9%	1656 13.8%	79 1.3%	43 2.4%	660 16.3%	648 18.0%	1054 15.5%	5700 10.1%
	Missing	8 0.1%	10 0.3%	560 12.3%	26 0.6%	409 3.4%	3 0.0%	19 1.1%	98 2.4%	32 0.9%	258 3.8%	1423 2.5%
	NA	4897 50.2%	1762 58.2%	0	0	0	3458 57.1%	1013 57.2%	0	0	0	11,130 19.8%
**Education**	Low	4785 49.0%	1450 47.9%	1940 42.7%	1875 40.5%	4524 37.7%	3143 51.9%	873 49.3%	1289 31.8%	1086 30.1%	2259 33.2%	23,224 41.3%
	Intermediate	2895 29.7%	910 30.1%	1688 37.2%	1517 32.8%	3692 30.7%	1586 26.2%	424 23.9%	1705 42.1%	1540 42.7%	2313 34.0%	18,270 32.5%
	High	1960 20.1%	611 20.2%	894 19.7%	1200 25.9%	3690 30.7%	1272 21.0%	398 22.5%	1024 25.3%	941 26.1%	2158 31.7%	14,148 25.2%
	Missing	120 1.2%	54 1.8%	19 0.4%	37 0.8%	102 0.8%	54 0.9%	77 4.3%	30 0.7%	37 1.0%	81 1.2%	611 1.1%
**Parity**	Yes	7325 75.2%	2179 73.1%	2787 61.7%	1610 59.3%	452 53.2%	5212 86.4%	1487 85.7%	3330 82.9%	2026 84.3%	474 82.6%	26,882 75.6%
	No	2413 24.8%	803 26.9%	1731 38.3%	1103 40.7%	397 46.8%	821 13.6%	249 14.3%	688 17.1%	378 15.7%	100 17.4%	8683 24.4%
	Missing	22 0.2%	43 1.4%	23 0.5%	11 0.2%	6 0.0%	22 0.4%	36 2.0%	30 0.7%	8 0.2%	4 0.1%	205 0.4%
	NA	0	0	0	1905 41.2%	11,153 92.9%	0	0	0	1192 33.1%	6233 91.5%	20,483 36.4%
**Physical activity**	Vigorous	618 12.7%	475 15.9%	1012 22.8%	1258 27.4%	3699 31.6%	358 10.5%	228 13.1%	640 16.0%	714 20.0%	1687 25.3%	10,689 22.3%
	Moderately active	2205 45.4%	1536 51.4%	2384 53.8%	2275 49.6%	5370 45.8%	1434 42.2%	892 51.4%	2758 69.0%	2202 61.6%	3533 53.0%	24,589 51.3%
	Inactive	2036 41.9%	977 32.7%	1036 23.4%	1058 23.0%	2648 22.6%	1604 47.2%	616 35.5%	600 15.0%	656 18.4%	1451 21.8%	12,682 26.4%
	Missing	38 0.4%	37 1.2%	109 2.4%	38 0.8%	291 2.4%	62 1.0%	36 2.0%	50 1.2%	32 0.9%	140 2.1%	833 1.5%
	NA	4863 49.8%	0	0	0	0	2597 42.9%	0	0	0	0	7460 13.3%
**BMI**	< 25	6950 71.2%	2103 69.5%	2488 54.8%	2901 62.7%	7701 64.1%	2629 43.4%	823 46.4%	2103 52.0%	1840 51.1%	3333 48.9%	32,871 58.4%
	25-29.9	2008 20.6%	658 21.8%	872 19.2%	1138 24.6%	2528 21.1%	2339 38.6%	653 36.9%	1195 29.5%	1123 31.2%	2033 29.8%	14,547 25.9%
	≥ 30	611 6.3%	205 6.8%	340 7.5%	557 12.0%	1454 12.1%	1001 16.5%	277 15.6%	640 15.8%	607 16.8%	1235 18.1%	6927 12.3%
	Missing	191 2.0%	59 2.0%	841 18.5%	33 0.7%	325 2.7%	86 1.4%	19 1.1%	110 2.7%	34 0.9%	210 3.1%	1908 3.4%
**IUD**	Never use	0	0	3746 82.5%	792 17.1%	9006 75 − 0%	0	0	2176 53.8%	467 13.0%	3784 55.6%	19,971 35.5%
	Ever use	0	0	286 6.3%	124 2.7%	1580 13.2%	0	0	341 8.4%	215 6.0%	2066 30.3%	4612 8.2%
	Missing	0	0	23 0.5%	12 0.3%	1422 11.8%	0	0	30 0.7%	6 0.2%	961 14.1%	2454 4.4%
	NA	9760 100%	3025 100%	486 10.7%	3701 80.0%	0	6055 100%	1772 100%	1501 37.1%	2916 80.9%	0	29,216 51.9%
**Hormonal contraceptives other than the IUD**	Never use	4224 43.3%	2679 88.6%	1315 29.0%	124 2.7%	6569 54.7%	3380 55.8%	1737 98.0%	1199 29.6%	143 4.0%	4816 70.7%	26,186 46.6%
	Ever use	673 6.9%	346 11.4%	2721 59.9%	792 17.1%	4020 33.5%	78 1.3%	35 2.0%	1329 32.8%	540 15.0%	1033 15.2%	1156720.6%
	Missing	0	0	19 0.4%	12 0.3%	1419 11.8%	0	0	19 0.5%	5 0.1%	962 14.1%	2436 4.3%
	NA	4863 49.8%	0	486 10.7%	3701 80.0%	0	2597 42.9%	0	1501 37.1%	2916 80.9%	0	16,064 28.6%
**Cohort**	ATH1	0	0	0	1905 41.2%	0	0	0	0	1192 33.1%	0	3097 5.5%
	ATH2	0	0	0	0	11,153 92.9%	0	0	0	0	6233 91.5%	17,386 30.9%
	FMCF	3905 40%	0	0	0	0	1802 29.8%	0	0	0	0	5707 10.1%
	Finrisk	4897 50.2%	3025 100%	2558 56.3%	2485 53.7%	855 7.1%	3458 57.1%	1772 100%	1690 41.7%	1691 46.9%	578 8.5%	23,009 40.9%
	H2000	0	0	1497 33.0%	0	0	0	0	857 21.2%	0	0	2354 4.2%
	HHS	0	0	486 10.7%	239 5.2%	0	0	0	1501 37.1%	721 20.0%	0	2947 5.2%
	MFS	958 9.8%	0	0	0	0	795 13.1%	0	0	0	0	1753 3.1%

Between 1972 and 2015, the age-standardized incidence of EOBC in overweight women aged 18–49 and 40–49 years increased by 3.4% (95% CI 0.0–6.9%,) and 4.0% (95% CI 0.5–7.7%) per year, respectively (Table 2). An increasing trend in EOBC incidence was observed in women engaging in moderate physical activity. The respective average annual increases were 2.4% (95% CI 0.0–4.9%,) among women aged 18–49 years and 2.9% (95% CI 0.3–5.6%) among women aged 40–49 years. Additionally, the incidence of EOBC increased by 3.3% (95% CI 0.1% to 6.6) per year among current smokers in the age group 40–49 years.

**Table 2:** Number of EOBCs, overall and risk factor stratified AAPC in incidence rates of EOBC during 1972–2015

Age group	18–39		40–49		18–49		
	**AAPC (%)**	**95% CI**	**AAPC**	**95%CI**	**AAPC**	**95% CI**	**Cancers**
Risk factor							
Smoking							
Never	-4.2%	-10.7-2.8%	1.2%	-1.0-3.4%	0.8%	-1.3-2.9%	196
Former	5.1%	-2.5-13.4%	-1.4%	-5.0-2.4%	0.6%	-2.7-4.0%	77
Current	-3.6%	-10.4-3.9%	3.3%	0.1–6.6%	2.3%	-0.5-5.3%	121
Alcohol consumption							
No	-8.5%	-23.4-9.2%	0.5%	-4.3-5.6%	-0.6%	-5.0-4.1%	40
Moderate	-1.5%	-6.0-3.3%	1.0%	-1.5-3.5%	0.8%	-1.4-3.0%	201
Heavy	-0.5%	-12.1-12.8%	1.4%	-4.6-7.7%	2.0%	-3.4-7.8%	34
Education							
Low	-2.6%	-9.0-4.2%	1.6%	-1.0-4.2%	1.3%	-1.1-3.7%	147
Intermediate	0.4%	-5.7-6.8%	2.2%	-0.5-5.1%	2.4%	-0.1-5.0%	153
High	-3.1%	-11.9-6.6%	0.0%	-3.2-3.4%	-0.2%	-3.2–2.9%	92
Physical activity							
Low	0.2%	-7.7-8.9%	1.5%	-3.0-6.2%	1.7%	-2.3-5.9%	62
Moderate	-1.1%	-7.0-5.1%	2.9%	0.3–5.6%	2.4%	0-4.9%	158
Vigorous	0.6%	-8.0-10.0%	-1.0%	-4.0-2.2%	-0.7%	-3.6-2.3%	97
BMI							
Normal	-2.1%	-6.6-2.5%	1.2%	-0.8-3.2%	1.1%	-0.7-2.9%	282
Overweight	-5.1%	-14.6-5.5%	4.0%	0.5–7.7%	3.4%	0-6.9%	80
Obese	19.1%	-8.0-53.9%	-0.8%	-6.7-5.4%	0.7%	-4.8–6.6%	26
Hormonal contraceptives other than the IUD							
Never use	-0.6%	-5.5-4.4%	1.4%	-0.7-3.5%	1.1%	-0.8-3.0%	187
Ever use	3.7%	-7.2-15.9%	-0.1%	-5.1-5.2%	1.0%	-3.7-5.8%	60
Overall adjusted^a^	-1.4%	-5.3-2.6%	1.6%	-0.1-3.2%	1.4%	-0.1-2.9%	
Overall unadjusted	2.9%	0.8–4.9	1.3%	0.4–2.2%	1.6%	0.7–2.4%	

^a^Adjusted for age, period, smoking, alcohol use, BMI, education, physical activity, and use of hormonal contraceptives other than the IUD. Adjustment for variables with structural missing values (alcohol use, physical activity and use of hormonal contraceptives other than IUD) was partial, with information lacking for certain previously specified cohorts

No significant changes in the risk factor stratified age-standardized incidence trends were observed among women aged 18–39. Furthermore, we did not observe any significant changes in trends by alcohol use, educational level, and use of other hormonal contraceptives than the IUD in any age group. IUD users were not included in the analysis as the number of subjects in this group was small. Risk factor stratified period trends in age-standardized rates and hazard ratios for breast cancer are presented in Supplementary Tables 2 (ages 18–39), 3 (ages 40–49) and 4 (ages 18–49).

Effects of modifiable factors on cause-specific hazard ratios (HRs) for EOBC in women aged 18–49 years are presented in Table 3 and in Supplementary Tables 5 and 6 for women aged 18–39 and 40–49, respectively. Overweight (BMI 25–30 kg/m^2^) and obesity (BMI ≥ 30 kg/m^2^) were statistically significant protective factors for EOBC. Compared to women with BMI less than 25 kg/m^2^, overweight decreased the risk of EOBC by 25% (95% CI 0.6-1.0), while obesity decreased the risk by 41% (95% CI 0.4–0.9). Women with intermediate education had an elevated risk of EOBC (HR 1.3, 95% CI 1.0-1.6), whereas a high level of education did not increase the risk statistically significantly (HR 1.2, 95% CI 0.9–1.5). The use of hormonal contraception did not significantly affect the risk of EOBC.

**Table 3:** Number of EOBCs, person years, age-standardized rates (ASR) and hazard ratios by risk factors during 1972–2015

Risk factor	Value	Person years	Cancers	ASR	Univariate		Multivariate^b^	
					HR	95% CI	HR	95% CI
Smoking	Never	230,316	196	46.4	1.00	1.00–1.00	1.00	
	Former	77,151	77	57.5	1.12	0.86–1.47	1.12	0.85–1.47
	Current	129,560	121	54.0	1.13	0.90–1.42	1.15	0.90–1.46
Alcohol	No use	56,779	40	38.8	1.00	1.00–1.00	1.00	
	Moderate use	204,581	201	58.5	1.25	0.87–1.78	1.14	0.79–1.66
	Heavy use	31,195	34	61.0	1.34	0.83–2.15	1.18	0.72–1.94
Education	Low	196,950	147	42.1	1.00	1.00–1.00	1.00	
	Intermediate	142,023	153	59.7	1.32	1.05–1.66	1.29	1.02–1.63
	High	95,707	92	53.0	1.21	0.93–1.57	1.15	0.87–1.50
Parity	Yes	275,600	262	46.9	1.00	1.00–1.00	1.00	
	No	115,680	101	57.2	1.14	0.90–1.44	1.13	0.89–1.44
Physical activity	Vigorous	63,575	62	61.7	1.00	1.00–1.00	1.00	
	Moderately active	167,103	158	51.1	0.88	0.65–1.18	0.90	0.67–1.22
	Inactive	99,857	97	52.7	1.00	0.72–1.37	1.09	0.78–1.51
BMI	< 25 kg/m^2^	284,958	282	57.4	1.00	1.00–1.00	1.00	
	25–29.9 kg/m^2^	97,728	80	40.4	0.74	0.58–0.96	0.75	0.58–0.96
	≥ 30 kg/m^2^	37,751	26	32.4	0.59	0.40–0.89	0.59	0.39–0.89
Use of IUD	Never use	99,040	80	52.4	1.00	1.00–1.00	1.00	
	Ever use	14,005	15	97.2	1.04	0.60–1.82	1.16	0.65–2.06
Hormonal contraceptives other than the IUD	Never use	190,055	187	50.7	1.00	1.00–1.00	1.00	
	Ever use	85,541	60	46.5	0.75	0.55–1.02	0.75	0.52–1.07

^b^Adjusted for age, period, alcohol use, BMI, education, use of IUD, use of hormonal contraceptives other than the IUD, smoking, parity and physical activity. Adjustment for variables with structural missing values (alcohol use, parity, physical activity, use of IUD and hormonal contraceptives other than IUD) was partial, with information lacking for certain previously specified cohorts

We did not find statistically significant associations between any other risk factors and EOBC.

## Discussion

The incidence of EOBC increased among overweight and moderately physically active women during 1972–2015. In age-stratified analyses, the exposure-specific trends were not significant among women under the age of 40, whereas significantly increasing incidence trends were observed among 40–49-year-old current smokers, women with overweight, and those engaging in only moderate physical activity. Overall, overweight and obese women had a lower risk of EOBC, while intermediate education was associated with an increased risk.

To the best of our knowledge, there are no previous studies examining risk factor-specific incidence trends of EOBC. It is known that the overall incidence of EOBC has increased in all Nordic countries during 1972–2015 with almost identical age-standardized incidence rates observed in Finland, Sweden, and Norway [36]. Furthermore, the incidence trends among Finnish women aged < 50 years are similar to those observed in other very high HDI countries [37]. The close resemblance of incidence trends likely reflects the similarities in the prevalence of certain lifestyle-related risk factors in these countries.

There is previous evidence of a positive association between high education level and the risk of EOBC [12, 14]. In our study, the risk of EOBC was 29% higher in women with intermediate education compared to women with low level of education. A study by Braaten et al. including premenopausal women from Sweden and Norway demonstrated that the risk is attenuated when adjusting for other risk factors such as parity and age at first birth [14]. Thus, a high level of education is not considered an independent risk factor but can be used as an indicator of the presence of relevant risk factors, such as later age at first childbirth [14, 38]. We lacked information on the age at first birth of the subjects but similarly to the findings of Braaten et al. [14] we also observed a clear attenuation of the hazard ratios after adjusting for other risk factors. Furthermore, in Finland, the number of students attending higher education (universities and polytechnic schools) almost tripled during the 1960s and has been continuously increasing ever since [39]. Therefore, more women are exposed to risk factors accompanied by high level of education. Additionally, it is probable that the differences in health behavior among women with high and intermediate education have narrowed down and therefore, could explain why the most prominent difference was observed between women with low and intermediate education.

We also found a significant inverse association between early adulthood BMI and the risk of EOBC. Being overweight or obese decreased the risk by 25% and 41% compared to women with BMI < 25 kg/m^2^, respectively. The protective effect of a high adulthood body weight on EOBC is well supported by earlier literature [5, 15, 18]. It has been proposed that the mechanism underlying the association between body weight and breast cancer involves hormonal pathways as adipose tissue is a known mediator of the levels of circulating sex hormones [40]. Obese women have been reported to experience an increased number of anovulatory cycles [41], leading to reduced exposure to endogenous ovarian hormones. Also, adulthood body weight is known to have a crossover effect on breast cancer depending on the menopausal status [17, 42], which further suggests a hormonal background.

During 2010–2013, around 40% of Finnish women were users of any form of hormonal contraception and Finland had the highest rate of IUD use in the Nordic countries [43]. In our study population, there were 4,612 IUD users and 11,567 users of other forms of hormonal contraceptives, most of whom were likely to be oral contraceptive users [43]. Our study did not reveal any significant change in risk among users of IUD or other hormonal contraceptives. In contrast to our findings, earlier evidence has suggested that the use of oral hormonal contraception might be associated with an increase in risk of EOBC [21, 26] while the independent effect of hormonal IUD use has remained controversial [44, 45]. It has earlier been observed that the use of oral contraceptives is associated with the risk of EOBC in a dose-dependent manner, with preparations with lower hormonal concentration being related to a lower risk [46]. In Finland, the concentration of ethinylestradiol in combined oral contraceptives has decreased since the product was first introduced in 1962, and low-dose preparations (20–30 µg) are currently the most commonly used formulations [47]. In addition, the risk seems to be modified by several factors such as duration of use [21, 48], age at first use [21], and time since last use [21]. Independent effects of these variables were not available in our study and therefore, it cannot be concluded whether any of these factors would have explained the inconsistency between our results and previous literature.

Although parity has been described as a protective factor for EOBC [12, 49] we could not confirm this finding. However, it is known that later age at first birth attenuates the protective effect of parity [12, 49]. In a recent meta-analysis by Nelson et al., women who had their first child at the age of 30 or older exhibited a higher risk of breast cancer compared to a reference group of women aged 25 to 29, with the risk only slightly lower than that of nulliparous women [49]. In 2020, the average age at first birth in Finland was 29.7 years, which could explain the absence of the protective effect in our study [50].

The causes of the increased breast cancer incidence in young women are not yet fully understood. Commonly, early age at onset of cancer has been thought to be associated with either inherited genetic susceptibility or early exposure to environmental risk factors, or the combinatory effect of both. Due to the speed of the increase, the change is unlikely to be entirely of genetic background. In order to determine the possible role of environmental exposure it is of use to identify potential environmental risk factors and combine these findings with information on the prevalence trends of the identified factors.

Significant increases in average annual percentage change of incidence across all age groups were fully attenuated after adjusting for modifiable risk factors, highlighting their crucial role in EOBC incidence. Risk factor stratified incidence trends seemed to differ between women aged 18–39 and 40–49, suggesting the possibility of etiological disparities or varying susceptibility to the effects of risk factors depending on the age at diagnosis. In our study population, the incidence of breast cancer increased significantly among overweight, moderately physically active, and actively smoking 40–49-year-old women, which was not seen in 18–39-year-old women. Furthermore, significant heterogeneity in incidence trends was observed in the youngest age group, showing a positive trend among former smokers and a negative trend among never and current smokers.

Regarding the increase in the incidence of EOBC among overweight women, there are likely to be unmeasured environmental factors contributing to the increase. For example, even though overweight is associated with reduced risk of EOBC, overweight women might be more prone to the effects of other, especially hormonal, risk factors as the protective effect of body adiposity is probably exerted through a hormonal pathway [42] and therefore, might be interfered. Differences in especially hormonal factors within this group of women should be further examined in order to identify possible characteristics that may reduce the protective effect of adipose tissue. We were not able to detect a change in the incidence of EO-BC among women with obesity possibly due to the low number of incident cancers in that category.

Results of a recent meta-analysis suggest that smoking moderately increases the risk of breast cancer and is not limited to current smokers but also extends to women exposed to secondhand tobacco smoke [8]. The exact reason for the observed incidence increase among currently smoking women requires further inspection of this group of women. One area of interest is the changes in the types of tobacco products used, as the concentration of carcinogenic chemicals and the route of absorption vary between different products. One plausible explanation for our heterogeneous findings in the younger age group is that passive smoking has decreased markedly as smoking in shared indoor areas has been prohibited by law in Finland since the late 20th century [51]. This might explain the difference in incidence by smoking status: non-smokers in the earliest birth cohorts may have been exposed to a higher amount of environmental tobacco smoke and therefore, had a higher baseline risk of breast cancer. Regarding moderately physically active women, this large group is likely to be more heterogeneous than vigorously active or inactive women. We also acknowledge that measuring physical activity as leisure-time activity is affected by the physical strain of one’s job and the amount of household chores, which we did not measure. Therefore, measuring individual’s physical activity only as leisure-time activity might be imprecise. In the future, it would be of interest to analyze this group in more detail to identify the characteristics that have led to an increased risk of EOBC in these women.

Our findings confirm the increased risk of EOBC with increasing education level and decreased risk in overweight women. However, increasing incidence among overweight, currently smoking or moderately physically active 40–49-year-old women are novel findings. More research is needed to confirm if the association between these factors and EOBC is causal in nature. Besides contributing to understanding the possible role of lifestyle factors behind increasing EOBC numbers, identifying lifestyle risk factors for early-onset breast cancer can help develop risk evaluation tools specifically for younger women. Currently, most breast cancer risk assessment models give minimal consideration to lifestyle factors and focus on family history and reproductive factors. Risk assessment tools can be utilized by healthcare professionals to evaluate the need and frequency of risk-based screening in women.

There were a few limitations in our study. Information on risk factors was partly based on questionnaires and therefore, our risk estimates may be affected by the response bias or measurement errors. For example, heavy drinkers are more prone to underestimate the amount of alcohol they consume [52], which could bias the hazard ratios towards the null. The individual missing values are not likely to affect the results much as their proportion is small: 4.5% at highest. However, the proportion of structural missing (concerning alcohol use, parity, physical activity, IUD and other hormonal contraceptives) is larger. Structural missingness applies to all individuals irrespective of their exposure status. In the statistical model we included a separate category for the structural missing values and therefore their effect could not be adjusted for in all cohorts. For instance, partial adjustment for parity might result in higher estimated effect of education in the multivariate model. Also, the risk factor exposure was only measured at the baseline of each survey, which can lead to some degree of missclassification. Change in the prevalence of risk factors, especially if the prevalence has increased, can result in some of the unexposed individuals becoming exposed during follow-up and thus, might lead to underestimation of the effects of these risk factors. However, the measurement of exposure to risk factors remained consistent throughout the entire study period in all cohorts. For example, even though the societal perception of the definition of moderate alcohol consumption has probably changed over time, the measurement of alcohol consumption in the study, quantity-wise, has remained unchanged and thus, is not affected by the individuals’ perception of what constitutes moderate or heavy drinking.

The estimation of HRs or AAPCs were not adjusted for cohort, because the number of cancer cases is very limited and the analyses were already adjusted for age and calendar time as well as other covariates which we think are the most relevant ones in terms of cancer incidence. In addition, if we adjusted the trend estimation for cohort, the estimates of AAPCs would be mainly based on cohorts with long follow-up and information on cancer incidence in the most recent cohorts (such as the large ATH1 and ATH2) would be underutilised. Therefore the analysis of AAPC was adjusted for IUD and parity neither, because the former was not available for the earliest cohorts and the latter was not available for the most recent cohorts (the large ATH cohorts). Furthermore, we could not comprehensively retrieve information on some of the known reproductive risk factors and thus, were not able to include the effects of these potentially confounding factors in our multivariate model. Information on the subjects’ menopausal status was also not available. As a result, some of the subjects may have entered menopause or be going through perimenopause before they were diagnosed with breast cancer or during the follow-up. However, the impact of this on the results is likely to be relatively small as the median age at menopause in Finland is 51 years [53]. Finally, EO-BC is relatively rare and thus, the number of subjects in our study may not be large enough to detect all statistically significant associations, especially in the younger age group. Some participants may have been included in more than one cohort, but the number of such participants was likely very small and unlikely to impact the results.

Strengths of our study include a long follow-up time. Also, the METCA data can be considered to represent the Finnish population relatively well with relatively high participation rates in surveys (51–94%) [35]. In addition, the prevalence rates of risk factors in our study are similar to those reported in other large nationwide studies reporting risk factor prevalence in Finland. The proportions and prevalence trends for smoking [54], parity [55], physical activity [54] and alcohol use [56] are really similar to those reported in external datasets, whereas our subjects were slightly leaner [54], less educated [39] and less likely to have ever used hormonal contraceptives [43]. Additionally, we used multivariate models adjusting for selected risk factors and individual study effects in order to limit the effects of potential confounding factors in the analysis. Another major strength of our study is near to complete coverage of breast cancers in the registry data. According to a quality and coverage study, the Finnish Cancer Registry had 98% coverage for breast tumors during the period 2009–2013, of which 99.5% were morphologically verified [57]. The Finnish population has a relatively homogenous ethnic background with the vast majority being Caucasian. Therefore, the findings of this study are generalizable to populations that share a similar prevalence of risk factors, mainly including other Western or very high HDI countries.

In conclusion, the increase in breast cancer incidence was highest among women aged 40–49 who were overweight, currently smoking, or moderately physically active, while no change in breast cancer incidence by risk factors was found in women under 40 years of age. Our findings suggest a different type of cancer process in the young adult population and highlight the importance of lifestyle risk factors in EOBC. Furthermore, preventive measures should take place already at childhood or early adulthood.

## Electronic supplementary material

Below is the link to the electronic supplementary material.

Supplementary Material 1

## Data Availability

Permission to use administrative health data, underlying our study, from various register keepers can be applied from Findata, the Social and Health Data Permit Authority. Requests to access these data can be submitted to the Findata: https://findata.fi/en/.

## Abbreviations

AAPC: average annual percent change
ATBC: The Alpha-Tocopherol, Beta-Carotene Cancer Prevention Study
ATH: Regional Health and Well-being Study
BMI: body mass index
CI: confidence interval
EOBC: early-onset breast cancer
FMCF: The Finnish Mobile Clinic Health Examination Follow-up Survey
H2000: Health 2000 Survey
HBCS: Helsinki Birth Cohort Study
HHS: Helsinki Health Study
HR: hazard ratio
IUD: intrauterine device
METCA: Prospective METa Cohort Study of Cancer Burden in Finland
MFS: Mini-Finland Health Survey
SIR: standardized incidence ratio
