# Supplementary material for: Incidence trends of early-onset breast cancer by lifestyle risk factors
Source: BMC Cancer. 2025 Feb 21;25:326. doi: 10.1186/s12885-025-13730-y (PMC11846213; doi:10.1186/s12885-025-13730-y)
Supplement: Supplementary file 1 — Supplementary Material 1 [file 12885_2025_13730_MOESM1_ESM.docx]

**Supplementary Tables**

**Incidence trends of early-onset breast cancer by lifestyle risk factors**

Jasmiina N. J. Rantala, Karri Seppä, Johan G. Eriksson, Sirpa Heinävaara, Tommi Härkänen, Pekka Jousilahti, Paul Knekt, Satu Männistö, Ossi Rahkonen, Harri Rissanen, Eetu Mäkinen, Nea Malila, Heidi Ryynänen, Maarit A. Laaksonen, Sanna M. M. Heikkinen and Janne M. Pitkäniemi, METCA Study Group

**Corresponding author**: Jasmiina N. J. Rantala, Faculty of Medicine, University of Helsinki, Helsinki, Finland; Finnish Cancer Registry, Institute for Statistical and Epidemiological Cancer Research, Helsinki, Finland; E-mail: [jasmiina.rantala@cancer.fi](mailto:jasmiina.rantala@cancer.fi)

**Supplementary Table 1. Cancer cases in women aged 18-49 years in each period and cohort.**

|  |  | **Cancer cases** |
| --- | --- | --- |
| **Period** | 1972-1981 | 42 |
|  | 1982-1991 | 89 |
|  | 1992-2001 | 117 |
|  | 2002-2011 | 107 |
|  | 2012-2015 | 42 |
| **Cohort** | ATH1 | 16 |
|  | ATH2 | 17 |
|  | FMCF | 60 |
|  | Finrisk | 238 |
|  | H2000 | 17 |
|  | HHS | 31 |
|  | MFS | 18 |

**Supplementary Table 2. Time trends in age standardized rates (ASR) and hazard ratios (HR) for breast cancer in women aged 18-39 years stratified by exposure to key modifiable risk factors (smoking, alcohol consumption, BMI, education level, physical activity and use of other hormonal contraception than the IUD).**

| Risk factor |  | |  | | | | |  | |  | | |  | | | | | | |  | |  |  |  |  |  |
| --- | --- | --- | --- | --- | --- | --- | --- | --- | --- | --- | --- | --- | --- | --- | --- | --- | --- | --- | --- | --- | --- | --- | --- | --- | --- | --- |
|  | **No** | | | | | | **Former** | | | | | | | | **Current** | | | | | |  |  |  |  |  |  |
|  | **Period** | **N per year** | | **ASR** | **HR** | **95% CI** | **Period** | | **N per year** | | **ASR** | **HR** | | **95% CI** | **Period** | **N per year** | **ASR** | **HR** | **95% CI** | |  |  |  |  |  |  |
| Smoking | 1972-1981 | 0.6 | | 21.8 | 6.9 | 0.43-109.93 | 1972-1981 | | 0 | | 0 | 0 | | - | 1972-1981 | 0.2 | 14.3 | 3.79 | 0.15-96.13 | |  |  |  |  |  |  |
|  | 1982-1991 | 0.3 | | 12.3 | 2.32 | 0.23-23.01 | 1982-1991 | | 0.1 | | 9.8 | 0.22 | | 0.01-4.83 | 1982-1991 | 0.6 | 32.4 | 6.53 | 0.38-111.14 | |  |  |  |  |  |  |
|  | 1992-2001 | 0.2 | | 19.9 | 1.18 | 0.18-7.96 | 1992-2001 | | 0.3 | | 48.5 | 0.77 | | 0.15-3.8 | 1992-2001 | 0.7 | 76.8 | 7.05 | 0.56-88.11 | |  |  |  |  |  |  |
|  | 2002-2011 | 0.7 | | 52 | 2.62 | 0.63-10.95 | 2002-2011 | | 0.5 | | 81.7 | 1.65 | | 0.37-7.43 | 2002-2011 | 0.2 | 15.5 | 1 | 0.09-11.34 | |  |  |  |  |  |  |
|  | 2012-2012 | 0.7 | | 18.6 | 1 | 1 | 2012-2015 | | 1 | | 61.3 | 1 | | 1 | 2012-2015 | 0.2 | 16.4 | 1 | 1 | |  |  |  |  |  |  |
|  | AAPC | - | | - | -4.16 | -10.67-2.82 | AAPC | | - | | - | 0.58% | | -2.49-13.35 | AAPC | - | - | -3.55% | -10.43-3.87 | |  |  |  |  |  |  |
|  | **No** | | | | | | **Moderate** | | | | | | | | **Heavy** | | | | | |  |  |  |  |  |  |
|  | **Period** | **N per year** | | **ASR** | **HR** | **95% CI** | **Period** | | **N per year** | | **ASR** | **HR** | | **95% CI** | **Period** | **N per year** | **ASR** | **HR** | **95% CI** | |  |  |  |  |  |  |
| Alcohol consumption | 1972-1981 | 0.4 | | 54.3 | 15.06 | - | 1972-1981 | | 0.2 | | 17.1 | 87793965.83 | | - | 1972-1981 | 0 | 0 | 0.16 | - | |  |  |  |  |  |  |
|  | 1982-1991 | 0.2 | | 27.3 | 5.98 | - | 1982-1991 | | 0.2 | | 12.3 | 1.29 | | 0.24-6.95 | 1982-1991 | 0 | 0 | 0 | - | |  |  |  |  |  |  |
|  | 1992-2001 | 0 | | 0 | 0 | - | 1992-2001 | | 1 | | 52.4 | 1.97 | | 0.65-5.91 | 1992-2001 | 0.2 | 76.6 | 1.72 | 0.18-16.42 | |  |  |  |  |  |  |
|  | 2002-2011 | 0 | | 0 | 0.97 | - | 2002-2011 | | 1.3 | | 53.8 | 2.06 | | 0.72-5.86 | 2002-2011 | 0.1 | 26 | 0.57 | 0.04-7.79 | |  |  |  |  |  |  |
|  | 2012-2015 | 0 | | 0 | 1 | 1 | 2012-2015 | | 1.5 | | 28.9 | 1 | | 1 | 2012-2015 | 0.5 | 66.4 | 1 | 1 | |  |  |  |  |  |  |
|  | AAPC | - | | - | -8.53 | -23.36-9.18 | AAPC | | - | | - | -1.48 | | -6.04-3.31 | AAPC | - | - | -0.45 | -12.13-12.78 | |  |  |  |  |  |  |
|  | **Normal** | | | | | | **Overweight** | | | | | | | | **Obese** | | | | | |  |  |  |  |  |  |
| BMI | **Period** | **N per year** | | **ASR** | **HR** | **95% CI** | **Period** | | **N per year** | | **ASR** | **HR** | | **95% CI** | **Period** | **N per year** | **ASR** | **HR** | **95% CI** | |  |  |  |  |  |  |
|  | 1972-1981 | 0.8 | | 25.4 | 3.41 | 0.59-19.82 | 1972-1981 | | 0 | | 0 | 6827929.98 | | - | 1972-1981 | 0 | 0 | 0 | - | |  |  |  |  |  |  |
|  | 1982-1991 | 0.9 | | 21.5 | 2.22 | 0.5-9.86 | 1982-1991 | | 0 | | 0 | 2.3 | | - | 1982-1991 | 0.1 | 38.2 | 0 | - | |  |  |  |  |  |  |
|  | 1992-2001 | 0.9 | | 48.3 | 1.92 | 0.62-5.94 | 1992-2001 | | 0.3 | | 65.8 | 411622606.64 | | - | 1992-2001 | 0 | 0 | 0 | - | |  |  |  |  |  |  |
|  | 2002-2011 | 0.9 | | 50 | 1.13 | 0.4-3.19 | 2002-2011 | | 0.4 | | 64.5 | 722031483.68 | | - | 2002-2011 | 0.1 | 38.5 | 1.4 | 0.06-32.39 | |  |  |  |  |  |  |
|  | 2012-2015 | 1.8 | | 42.4 | 1 | 1 | 2012-2015 | | 0 | | 0 | 1 | | 1 | 2012-2015 | 0.2 | 39.1 | 1 | 1 | |  |  |  |  |  |  |
|  | AAPC | - | | - | -2.14% | -6.58-2.51 | AAPC | | - | | - | -5.07 | | -14.56 -5.48 | AAPC | - | - | 19.07 | -7.89-53.91 | |  |  |  |  |  |  |
|  | **Low** | | | | | | **Middle** | | | | | | | | **High** | | | | | |  |  |  |  |  |  |
|  | **Period** | **N per year** | | **ASR** | **HR** | **95% CI** | **Period** | | **N per year** | | **ASR** | **HR** | | **95% CI** | **Period** | **N per year** | **ASR** | **HR** | **95% CI** | |  |  |  |  |  |  |
| Education | 1972-1981 | 0.3 | | 13.9 | 4.72 | 0.27-81.66 | 1972-1981 | | 0.2 | | 14 | 1.21 | | 0.09-16.97 | 1972-1981 | 0.3 | 32.9 | 3.87 | 0.1-142.87 | |  |  |  |  |  |  |
|  | 1982-1991 | 0.3 | | 12.2 | 2.47 | 0.24-25.32 | 1982-1991 | | 0.4 | | 22.1 | 1.6 | | 0.21-12.16 | 1982-1991 | 0.2 | 21.4 | 2.19 | 0.07-64.24 | |  |  |  |  |  |  |
|  | 1992-2001 | 0.6 | | 56.2 | 3.39 | 0.56-20.31 | 1992-2001 | | 0.4 | | 41.6 | 1.09 | | 0.24-4.89 | 1992-2001 | 0.2 | 38.4 | 4.59 | 0.37-56.44 | |  |  |  |  |  |  |
|  | 2002-2011 | 0.6 | | 40.8 | 2.16 | 0.42-11.17 | 2002-2011 | | 0.7 | | 67.7 | 1.85 | | 0.46-7.37 | 2002-2011 | 0.1 | 14.2 | 0.39 | 0.04-3.88 | |  |  |  |  |  |  |
|  | 2012-2015 | 0.5 | | 20.6 | 1 | 1 | 2012-2015 | | 0.9 | | 45.2 | 1 | | 1 | 2012-2015 | 0.7 | 38.3 | 1 | 1 | |  |  |  |  |  |  |
|  | AAPC | - | | - | -2.6 | -8.98-4.22 | AAPC | | - | | - | 0.36 | | -5.69-6.8 | AAPC | - | - | -3.14 | -11.94-6.55 | |  |  |  |  |  |  |
|  | **Low** | | | | | | **Moderate** | | | | | | | | **High** | | | | | |  |  |  |  |  |  |
|  | **Period** | **N per year** | | **ASR** | **HR** | **95% CI** | **Period** | | **N per year** | | **ASR** | **HR** | | **95% CI** | **Period** | **N per year** | **ASR** | HR | 95% CI | |  |  |  |  |  |  |
| Physical activity | 1972-1981 | 0 | | 0 | 0 | - | 1972-1981 | | 0 | | 0 | 0 | | - | 1972-1981 | 0.2 | 16.9 | 0.84 | 0.02-34.32 | |  |  |  |  |  |  |
|  | 1982-1991 | 0.1 | | 19 | 1.03 | 0.04-29.99 | 1982-1991 | | 0.5 | | 31.9 | 2.65 | | 0.42-16.57 | 1982-1991 | 0.2 | 16.6 | 0.86 | 0.03-25.29 | |  |  |  |  |  |  |
|  | 1992-2001 | 0.4 | | 73.5 | 2.49 | 0.46-13.51 | 1992-2001 | | 0.5 | | 43.8 | 1.93 | | 0.4-9.39 | 1992-2001 | 0.3 | 46.4 | 2.2 | 0.19-24.91 | |  |  |  |  |  |  |
|  | 2002-2011 | 0.6 | | 66.3 | 1.01 | 0.27-3.71 | 2002-2011 | | 0.5 | | 32.7 | 2.34 | | 0.44-12.35 | 2002-2011 | 0.3 | 38.8 | 2.22 | 0.21-23.73 | |  |  |  |  |  |  |
|  | 2012-2015 | 0.9 | | 50.1 | 1 | 1 | 2012-2015 | | 0.6 | | 21.3 | 1 | | 1 | 2012-2015 | 0.2 | 19.9 | 1 | 1 | |  |  |  |  |  |  |
|  | AAPC | - | | - | 0.24 | -7.7-8.88 | AAPC | | - | | - | -1.12 | | -6.98-5.12 | AAPC | - | - | 0.64% | -7.79-10.04 | |  |  |  |  |  |  |
|  | **Non user** | | | | | | **User** | | | | | | | |  |  |  |  |  |  |  |  |  |  |  |  |
| Other hormonal contraceptives than the IUD | **Period** | **N per year** | | **ASR** | **HR** | **95% CI** | **Period** | | **N per year** | | **ASR** | **HR** | | **95% CI** |  |  |  |  |  |  |  |  |  |  |  |  |
|  | 1972-1981 | 0.2 | | 8.9 | 0.77 | 0.08-7.12 | 1972-1981 | | 0 | | 0 | 0.07 | | - |  |  |  |  |  |  |  |  |  |  |  |  |
|  | 1982-1991 | 0.8 | | 30.6 | 1.57 | 0.32-7.78 | 1982-1991 | | 0 | | 0 | 0 | | - |  |  |  |  |  |  |  |  |  |  |  |  |
|  | 1992-2001 | 1 | | 78.8 | 1.85 | 0.49-7 | 1992-2001 | | 0.2 | | 21.1 | 0.7 | | 0.09-5.24 |  |  |  |  |  |  |  |  |  |  |  |  |
|  | 2002-2011 | 0.1 | | 30.4 | 1.01 | 0.1-10.03 | 2002-2011 | | 0.4 | | 22.3 | 1.08 | | 0.18-6.66 |  |  |  |  |  |  |  |  |  |  |  |  |
|  | 2012-2015 | 0.7 | | 32 | 1 | 1 | 2012-2015 | | 0.4 | | 18.8 | 1 | | 1 |  |  |  |  |  |  |  |  |  |  |  |  |
|  | AAPC (%) | - | | - | -0.64 | -5.46-4.43 | AAPC (%) | | - | | - | 3.7 | | -7.18-15.85 |  |  |  |  |  |  |  |  |  |  |  |  |

**Supplementary Table 3. Time trends in age standardized rates (ASR) and hazard ratios (HR) for breast cancer in women aged 40-49 years stratified by exposure to key modifiable risk factors (smoking, alcohol consumption, BMI, education level, physical activity and use of other hormonal contraception than the IUD).**

| Risk factor |  | | | | | | |  | | | |  | | | |  | | |  | | | | | | | |  | | | | | | | | | | |  | | | | | | | | |  | | | | | |  |  |  |  |  |
| --- | --- | --- | --- | --- | --- | --- | --- | --- | --- | --- | --- | --- | --- | --- | --- | --- | --- | --- | --- | --- | --- | --- | --- | --- | --- | --- | --- | --- | --- | --- | --- | --- | --- | --- | --- | --- | --- | --- | --- | --- | --- | --- | --- | --- | --- | --- | --- | --- | --- | --- | --- | --- | --- | --- | --- | --- | --- |
| Smoking | **No** | | | | | | | | | | | | | | | | | **Former** | | | | | | | | | | | | | **Current** | | | | | | | | | | | | | | | | | | | | |  |  |  |  |  |  |
|  | Period | | N per year | | | ASR | | | | HR | | | | | 95% CI | | | Period | | | | N per year | | ASR | | HR | | | | 95% CI | Period | | | N per year | | | ASR | | | | HR | | | | 95% CI | | | | | | | | | | | | |
|  | 1972-1981 | | 2.4 | | | 82.1 | | | | 0.83 | | | | | 0.35-1.95 | | | 1972-1981 | | | | 0.4 | | 177.7 | | 2.17 | | | | 0.5-9.46 | 1972-1981 | | | 0.6 | | | 88.6 | | | | 0.41 | | | | 0.12-1.39 | | | | | | | | | | | | |
|  | 1982-1991 | | 4.6 | | | 105.2 | | | | 0.99 | | | | | 0.45-2.18 | | | 1982-1991 | | | | 1.3 | | 154.2 | | 1.83 | | | | 0.56-5.95 | 1982-1991 | | | 2 | | | 118.4 | | | | 0.51 | | | | 0.18-1.39 | | | | | | | | | | | | |
|  | 1992-2001 | | 4.4 | | | 152.8 | | | | 1.32 | | | | | 0.66-2.65 | | | 1992-2001 | | | | 2.1 | | 172.1 | | 1.8 | | | | 0.67-4.82 | 1992-2001 | | | 4 | | | 208.8 | | | | 0.9 | | | | 0.38-2.14 | | | | | | | | | | | | |
|  | 2002-2011 | | 4.6 | | | 174.6 | | | | 1.53 | | | | | 0.83-2.82 | | | 2002-2011 | | | | 1.9 | | 129.7 | | 1.3 | | | | 0.53-3.17 | 2002-2011 | | | 2.6 | | | 125.5 | | | | 0.53 | | | | 0.25-1.13 | | | | | | | | | | | | |
|  | 2012-2012 | | 3.8 | | | 125.5 | | | | 1 | | | | | 1 | | | 2012-2015 | | | | 1.7 | | 110.5 | | 1 | | | | 1 | 2012-2015 | | | 2.7 | | | 163.6 | | | | 1 | | | | 1 | | | | | | | | | | | | |
|  | AAPC | | - | | | - | | | | 1.19% | | | | | -1.02-3.44 | | | AAPC | | | | - | | - | | 1.37% | | | | -4.98-2.39 | AAPC | | | - | | | - | | | | 3.25% | | | | 0.05-6.55 | | | | | | | | | | | | |
| Alcohol consumption | **No** | | | | | | | | | | | | | | | | | **Moderate** | | | | | | | | | | | | | **Heavy** | | | | | | | | | | | | | | | | | | | |  |  |  |  |  |  |  |
|  | **Period** | | **N per year** | | | **ASR** | | | | **HR** | | | | | **95% CI** | | | **Period** | | | | **N per year** | | **ASR** | | **HR** | | | | **95% CI** | **Period** | | | **N per year** | | | **ASR** | | | | **HR** | | | | **95% CI** | | | | | | | | | | | | |
|  | 1972-1981 | | 0.4 | | | 51.6 | | | | 0.8 | | | | | 0.1-6.53 | | | 1972-1981 | | | | 0.4 | | 68.8 | | 0.53 | | | | 0.16-1.76 | 1972-1981 | | | 0.2 | | | 422.2 | | | | 1.84 | | | | 0.2-16.6 | | | | | | | | | | | | |
|  | 1982-1991 | | 1.3 | | | 109.4 | | | | 1.55 | | | | | 0.24-10.19 | | | 1982-1991 | | | | 2 | | 116.8 | | 0.92 | | | | 0.43-1.98 | 1982-1991 | | | 0.3 | | | 169.6 | | | | 0.88 | | | | 0.13-6.14 | | | | | | | | | | | | |
|  | 1992-2001 | | 1 | | | 126 | | | | 1.63 | | | | | 0.27-9.73 | | | 1992-2001 | | | | 4.6 | | 169.8 | | 1.28 | | | | 0.73-2.23 | 1992-2001 | | | 0.6 | | | 154.9 | | | | 0.73 | | | | 0.2-2.76 | | | | | | | | | | | | |
|  | 2002-2011 | | 0.4 | | | 111 | | | | 0.99 | | | | | 0.2-4.95 | | | 2002-2011 | | | | 7.3 | | 155.7 | | 1.11 | | | | 0.69-1.79 | 2002-2011 | | | 1.3 | | | 155.8 | | | | 0.85 | | | | 0.27-2.67 | | | | | | | | | | | | |
|  | 2012-2015 | | 0.7 | | | 127.2 | | | | 1 | | | | | 1 | | | 2012-2015 | | | | 6.2 | | 138.6 | | 1 | | | | 1 | 2012-2015 | | | 1.2 | | | 135.4 | | | | 1 | | | | 1 | | | | | | | | | | | | |
|  | AAPC | | - | | | - | | | | 0.54% | | | | | -4.26- 5.59 | | | AAPC | | | | - | | - | | 0.97% | | | | -1.5-3.5 | AAPC | | | - | | | - | | | | 1.38% | | | | -4.58-7.71 | | | | | | | | | | | | |
| BMI | **Normal** | | | | | | | | | | | | | | | | | **Overweight** | | | | | | | | | | | | | **Obese** | | | | | | | | | | | | | | | | | | | |  |  |  |  |  |  |  |
|  | **Period** | | **N per year** | | | **ASR** | | | | **HR** | | | | | **95% CI** | | | **Period** | | | | **N per year** | | **ASR** | | **HR** | | | **95% CI** | | **Period** | | **N per year** | | | **ASR** | | | | **HR** | | | | **95% CI** | | | | | | | | | | | | | |
|  | 1972-1981 | | 2 | | | 103.7 | | | | 0.9 | | | | | 0.41-1.99 | | | 1972-1981 | | | | 1 | | 75.6 | | 0.36 | | | 0.11-1.22 | | 1972-1981 | | 0.3 | | | 58.3 | | | | 1.22 | | | | 0.13-11.8 | | | | | | | | | | | | | |
|  | 1982-1991 | | 5.8 | | | 127.1 | | | | 1.07 | | | | | 0.53-2.15 | | | 1982-1991 | | | | 1.5 | | 88.1 | | 0.42 | | | 0.14-1.31 | | 1982-1991 | | 0.5 | | | 91 | | | | 1.7 | | | | 0.25-11.69 | | | | | | | | | | | | | |
|  | 1992-2001 | | 7.6 | | | 177.2 | | | | 1.41 | | | | | 0.75-2.64 | | | 1992-2001 | | | | 2.1 | | 177.5 | | 0.9 | | | 0.36-2.23 | | 1992-2001 | | 0.5 | | | 106.9 | | | | 1.4 | | | | 0.31-6.4 | | | | | | | | | | | | | |
|  | 2002-2011 | | 7 | | | 182 | | | | 1.49 | | | | | 0.85-2.61 | | | 2002-2011 | | | | 1.5 | | 99.7 | | 0.45 | | | 0.2-1.04 | | 2002-2011 | | 0.6 | | | 87.7 | | | | 0.79 | | | | 0.19-3.22 | | | | | | | | | | | | | |
|  | 2012-2015 | | 4 | | | 119.9 | | | | 1 | | | | | 1 | | | 2012-2015 | | | | 3 | | 178.3 | | 1 | | | 1 | | 2012-2015 | | 1 | | | 102.3 | | | | 1 | | | | 1 | | | | | | | | | | | | | |
|  | AAPC | | - | | | - | | | | 1.19% | | | | | -0.76-3.17 | | | AAPC | | | | - | | - | | 4.03% | | | 0.45-7.74 | | AAPC | | - | | | - | | | | 0.84% | | | | -6.74-5.44 | | | | | | | | | | | | | |
| Education | **Low** | | | | | | | | | | | | | | | | **Medium** | | | | | | | | | | | | | | **High** | | | | | | | | | | | | | | | | | |  |  |  |  |  |  |  |  |  |
|  | **Period** | **N per year** | | | **ASR** | | | | **HR** | | | | | **95% CI** | | | **Period** | | | **N per year** | | | **ASR** | | **HR** | | | **95% CI** | | | **Period** | **N per year** | | | **ASR** | | | | **HR** | | | **95% CI** | | | | | |  |  |  |  |  |  |  |  |  |  |
|  | 1972-1981 | 1.7 | | | 82.1 | | | | 0.64 | | | | | 0.23-1.77 | | | 1972-1981 | | | | 0.7 | | 71.6 | | 0.56 | | | 0.19-1.67 | | | 1972-1981 | 1 | | | 129.8 | | | | 1.31 | | | 0.38-4.48 | | | | | |  |  |  |  |  |  |  |  |  |  |
|  | 1982-1991 | 3.3 | | | 95 | | | | 0.72 | | | | | 0.28-1.81 | | | 1982-1991 | | | | 2.6 | | 130.5 | | 0.95 | | | 0.4-2.26 | | | 1982-1991 | 1.9 | | | 139.7 | | | | 1.24 | | | 0.41-3.8 | | | | | |  |  |  |  |  |  |  |  |  |  |
|  | 1992-2001 | 4.3 | | | 159.7 | | | | 1.25 | | | | | 0.54-2.87 | | | 1992-2001 | | | | 4 | | 206.2 | | 1.26 | | | 0.62-2.59 | | | 1992-2001 | 2 | | | 153.9 | | | | 1.26 | | | 0.48-3.29 | | | | | |  |  |  |  |  |  |  |  |  |  |
|  | 2002-2011 | 2.4 | | | 120.3 | | | | 1.17 | | | | | 0.56-2.47 | | | 2002-2011 | | | | 4.5 | | 166.8 | | 0.89 | | | 0.46-1.69 | | | 2002-2011 | 2.3 | | | 161.9 | | | | 1.17 | | | 0.51-2.66 | | | | | |  |  |  |  |  |  |  |  |  |  |
|  | 2012-2015 | 2.6 | | | 113.3 | | | | 1 | | | | | 1 | | | 2012-2015 | | | | 3.5 | | 160 | | 1 | | | 1 | | | 2012-2015 | 2.3 | | | 126.3 | | | | 1 | | | 1 | | | | | |  |  |  |  |  |  |  |  |  |  |
|  | AAPC | - | | | - | | | | 1.57% | | | | | -0.99-4.21 | | | AAPC | | | | - | | - | | 2.24% | | | -0.53-5.1 | | | AAPC | - | | | - | | | | 0.04% | | | | -3.19- 3.37 | | | | | | |  |  |  |  |  |  |  |  |
|  | **Low** | | | | | | | | | | | | | | | | **Moderate** | | | | | | | | | | | | | | **Heavy** | | | | | | | | | | | | | | |  |  |  |  |  |  |  |  |  |  |  |  |
| Physical activity | **Period** | **N per year** | | | **ASR** | | | | **HR** | | | | | **95% CI** | | | **Period** | | | **N per year** | | | **ASR** | | **HR** | | | **95% CI** | | | **Period** | **N per year** | | | **ASR** | | | | **HR** | | | **95% CI** | | | | | | | |  |  |  |  |  |  |  |  |
|  | 1972-1981 | 0.3 | | | 102.4 | | | | 0.71 | | | | | 0.13-3.81 | | | 1972-1981 | | | 0.9 | | | 91.9 | | 0.43 | | | 0.16-1.15 | | | 1972-1981 | 1.2 | | | 109.7 | | | | 2.21 | | | 0.59-8.22 | | | | | | | |  |  |  |  |  |  |  |  |
|  | 1982-1991 | 0.5 | | | 88 | | | | 0.53 | | | | | 0.12-2.31 | | | 1982-1991 | | | 1.9 | | | 94.4 | | 0.46 | | | 0.2-1.06 | | | 1982-1991 | 2.3 | | | 141.1 | | | | 2.38 | | | 0.71-7.99 | | | | | | | |  |  |  |  |  |  |  |  |
|  | 1992-2001 | 1.3 | | | 188.8 | | | | 1.26 | | | | | 0.45-3.59 | | | 1992-2001 | | | 3.9 | | | 170.5 | | 0.9 | | | 0.47-1.71 | | | 1992-2001 | 2.5 | | | 199 | | | | 2.7 | | | 0.92-7.94 | | | | | | | |  |  |  |  |  |  |  |  |
|  | 2002-2011 | 1.8 | | | 148.9 | | | | 1.09 | | | | | 0.46-2.58 | | | 2002-2011 | | | 5.3 | | | 145.6 | | 0.86 | | | 0.49-1.51 | | | 2002-2011 | 2.1 | | | 181.2 | | | | 1.95 | | | 0.73-5.23 | | | | | | | |  |  |  |  |  |  |  |  |
|  | 2012-2015 | 2 | | | 131.5 | | | | 1 | | | | | 1 | | | 2012-2015 | | | 5 | | | 155.7 | | 1 | | | 1 | | | 2012-2015 | 1.2 | | | 86.9 | | | | 1 | | | 1 | | | | | | | |  |  |  |  |  |  |  |  |
|  | AAPC | - | | | - | | | | 1.48% | | | | | -3.03-6.21 | | | AAPC | | | - | | | - | | 2.93% | | | 0.29-5.63 | | | AAPC | - | | | - | | | | 0.95% | | | -4.02-2.22 | | | | | | | |  |  |  |  |  |  |  |  |
|  | **Non user** | | | | | | | | | | | | | | | | **User** | | | | | | | | | | | | | |  |  |  |  |  |  |  |  |  |  |  |  |  |  |  |  |  |  |  |  |  |  |  |  |  |  |  |
| Other hormonal contraceptives than the IUD | **Period** | | | **N per year** | | | **ASR** | | | | **HR** | | **95% CI** | | | | **Period** | | | | **N per year** | | | **ASR** | | **HR** | | | **95% CI** | |  |  |  |  |  |  |  |  |  |  |  |  |  |  |  |  |  |  |  |  |  |  |  |  |  |  |  |
|  | 1972-1981 | | | 2.4 | | | 105.3 | | | | 0.83 | | 0.36-1.9 | | | | 1972-1981 | | | | 0 | | | 0 | | 0 | | | - | |  |  |  |  |  |  |  |  |  |  |  |  |  |  |  |  |  |  |  |  |  |  |  |  |  |  |  |
|  | 1982-1991 | | | 4.5 | | | 119.2 | | | | 0.86 | | 0.4-1.84 | | | | 1982-1991 | | | | 0.2 | | | 45.3 | | 0.37 | | | 0.06-2.24 | |  |  |  |  |  |  |  |  |  |  |  |  |  |  |  |  |  |  |  |  |  |  |  |  |  |  |  |
|  | 1992-2001 | | | 5.8 | | | 173.5 | | | | 1.25 | | 0.63-2.45 | | | | 1992-2001 | | | | 1.6 | | | 200.9 | | 1.51 | | | 0.64-3.54 | |  |  |  |  |  |  |  |  |  |  |  |  |  |  |  |  |  |  |  |  |  |  |  |  |  |  |  |
|  | 2002-2011 | | | 2.4 | | | 175.4 | | | | 1.3 | | 0.64-2.63 | | | | 2002-2011 | | | | 2.3 | | | 116.1 | | 0.8 | | | 0.39-1.66 | |  |  |  |  |  |  |  |  |  |  |  |  |  |  |  |  |  |  |  |  |  |  |  |  |  |  |  |
|  | 2012-2015 | | | 3 | | | 120.9 | | | | 1 | | 1 | | | | 2012-2015 | | | | 2.7 | | | 139.3 | | 1 | | | 1 | |  |  |  |  |  |  |  |  |  |  |  |  |  |  |  |  |  |  |  |  |  |  |  |  |  |  |  |
|  | AAPC | | | - | | | - | | | | 1.35% | | -0.7-3.45 | | | | AAPC | | | | - | | | - | | 0.06% | | | -5.05-5.18 | |  |  |  |  |  |  |  |  |  |  |  |  |  |  |  |  |  |  |  |  |  |  |  |  |  |  |  |

**Supplementary Table 4. Time trends in age standardized rates (ASR) and hazard ratios (HR) for breast cancer in women aged 18-49 years stratified by exposure to key modifiable risk factors (smoking, alcohol consumption, BMI, education level, physical activity and use of other hormonal contraception than the IUD).**

| Risk factor |  |  | | | | | |  | |  | | |  | | | | | |  | | |  |  |  |  |  |  |  |
| --- | --- | --- | --- | --- | --- | --- | --- | --- | --- | --- | --- | --- | --- | --- | --- | --- | --- | --- | --- | --- | --- | --- | --- | --- | --- | --- | --- | --- |
| Smoking | **No** | | | | | | **Former** | | | | | | | | **Current** | | | | |  |  |  |  |  |  |  |  |  |
|  | **Period** | | **N per year** | **ASR** | **HR** | **95% CI** | **Period** | | **N per year** | | **ASR** | **HR** | | **95% CI** | **Period** | **N per year** | **ASR** | **HR** | | **95% CI** |  |  |  |  |  |  |  |  |
|  | 1972-1981 | | 3 | 58.7 | 0.94 | 0.42-2.09 | 1972-1981 | | 0.4 | | 100.2 | 1.07 | | 0.27-4.25 | 1972-1981 | 0.8 | 54.2 | 0.53 | | 0.17-1.6 |  |  |  |  |  |  |  |  |
|  | 1982-1991 | | 4.9 | 69 | 1.03 | 0.49-2.16 | 1982-1991 | | 1.4 | | 91.2 | 1.2 | | 0.43-3.38 | 1982-1991 | 2.6 | 78.6 | 0.76 | | 0.3-1.9 |  |  |  |  |  |  |  |  |
|  | 1992-2001 | | 4.6 | 101.1 | 1.37 | 0.72-2.62 | 1992-2001 | | 2.4 | | 118.2 | 1.44 | | 0.63-3.28 | 1992-2001 | 4.7 | 147.6 | 1.3 | | 0.6-2.82 |  |  |  |  |  |  |  |  |
|  | 2002-2011 | | 5.3 | 126.9 | 1.65 | 0.94-2.89 | 2002-2011 | | 2.4 | | 108.7 | 1.2 | | 0.57-2.5 | 2002-2011 | 2.8 | 74.6 | 0.6 | | 0.29-1.22 |  |  |  |  |  |  |  |  |
|  | 2012-2015 | | 4.5 | 83.9 | 1 | 1 | 2012-2015 | | 2.7 | | 89 | 1 | | 1 | 2012-2015 | 2.9 | 95.4 | 1 | | 1 |  |  |  |  |  |  |  |  |
|  | AAPC | | - | - | 0.79% | -1.29-2.92 | AAPC | | - | | - | 0.58% | | -2.74-4.02 | AAPC | - | - | 2.33% | | -0.54-5.28 |  |  |  |  |  |  |  |  |
| Alcohol consumption | **No** | | | | | | **Moderate** | | | | | | | | **Heavy** | | | | |  |  |  |  |  |  |  |  |  |
|  | **Period** | | **N per year** | **ASR** | **HR** | **95% CI** | **Period** | | **N per year** | | **ASR** | **HR** | | **95% CI** | **Period** | **N per year** | **ASR** | **HR** | | **95% CI** |  |  |  |  |  |  |  |  |
|  | 1972-1981 | | 0.8 | 52.7 | 1.39 | 0.19-9.98 | 1972-1981 | | 0.6 | | 46.2 | 0.62 | | 0.22-1.72 | 1972-1981 | 0.2 | 240.7 | 1.23 | | 0.15-10.23 |  |  |  |  |  |  |  |  |
|  | 1982-1991 | | 1.5 | 75.5 | 1.67 | 0.26-10.78 | 1982-1991 | | 2.2 | | 71.1 | 0.92 | | 0.46-1.86 | 1982-1991 | 0.3 | 96.7 | 0.7 | | 0.11-4.36 |  |  |  |  |  |  |  |  |
|  | 1992-2001 | | 1 | 74 | 1.74 | 0.29-10.32 | 1992-2001 | | 5.6 | | 118.5 | 1.43 | | 0.88-2.34 | 1992-2001 | 0.8 | 121.2 | 0.83 | | 0.27-2.55 |  |  |  |  |  |  |  |  |
|  | 2002-2011 | | 0.4 | 65.2 | 1.01 | 0.2-4.98 | 2002-2011 | | 8.6 | | 111.2 | 1.23 | | 0.8-1.89 | 2002-2011 | 1.4 | 100 | 0.75 | | 0.27-2.07 |  |  |  |  |  |  |  |  |
|  | 2012-2015 | | 0.7 | 74.7 | 1 | 1 | 2012-2015 | | 7.7 | | 90.6 | 1 | | 1 | 2012-2015 | 1.7 | 105.7 | 1 | | 1 |  |  |  |  |  |  |  |  |
|  | AAPC | | - | - | 0.56% | -5.02-4.11 | AAPC | | - | | - | 0.75% | | -1.44-2.98 | AAPC | - | - | 2.03% | | -3.42-7.79 |  |  |  |  |  |  |  |  |
| BMI | **Normal** | | | | | | **Overweight** | | | | | | | | **Obese** | | | | |  |  |  |  |  |  |  |  |  |
|  | **Period** | | **N per year** | **ASR** | **HR** | **95% CI** | **Period** | | **N per year** | | **ASR** | **HR** | | **95% CI** | **Period** | **N per year** | **ASR** | **HR** | | **95% CI** |  |  |  |  |  |  |  |  |
|  | 1972-1981 | | 2.8 | 69.3 | 0.94 | 0.47-1.88 | 1972-1981 | | 1 | | 49.6 | 0.42 | | 0.13-1.35 | 1972-1981 | 0.3 | 39.8 | 0.74 | | 0.09-6.26 |  |  |  |  |  |  |  |  |
|  | 1982-1991 | | 6.7 | 80.7 | 1.07 | 0.58-1.98 | 1982-1991 | | 1.5 | | 57.7 | 0.52 | | 0.18-1.52 | 1982-1991 | 0.6 | 74.3 | 1.33 | | 0.23-7.83 |  |  |  |  |  |  |  |  |
|  | 1992-2001 | | 8.5 | 120.6 | 1.46 | 0.85-2.5 | 1992-2001 | | 2.4 | | 139 | 1.22 | | 0.52-2.85 | 1992-2001 | 0.5 | 72.9 | 1.04 | | 0.26-4.13 |  |  |  |  |  |  |  |  |
|  | 2002-2011 | | 7.9 | 124 | 1.41 | 0.87-2.29 | 2002-2011 | | 1.9 | | 87.6 | 0.67 | | 0.31-1.46 | 2002-2011 | 0.7 | 72 | 0.83 | | 0.23-2.97 |  |  |  |  |  |  |  |  |
|  | 2012-2015 | | 5.8 | 85.9 | 1 | 1 | 2012-2015 | | 3 | | 116.9 | 1 | | 1 | 2012-2015 | 1.2 | 82.3 | 1 | | 1 |  |  |  |  |  |  |  |  |
|  | AAPC | | - | - | 1.05% | -0.73-2.87 | AAPC | | - | | - | 3.39% | | 0-6.9 | AAPC | - | - | 0.74% | | -4.79-6.59 |  |  |  |  |  |  |  |  |
| Education | **Low** | | | | | | **Middle** | | | | | | | | **High** | | | | |  |  |  |  |  |  |  |  |  |
|  | **Period** | | **N per year** | **ASR** | **HR** | **95% CI** | **Period** | | **N per year** | | **ASR** | **HR** | | **95% CI** | **Period** | **N per year** | **ASR** | **HR** | | **95% CI** |  |  |  |  |  |  |  |  |
|  | 1972-1981 | | 2 | 52.5 | 0.8 | 0.32-2.03 | 1972-1981 | | 0.9 | | 48.5 | 0.52 | | 0.19-1.39 | 1972-1981 | 1.3 | 89.8 | 1.4 | | 0.46-4.3 |  |  |  |  |  |  |  |  |
|  | 1982-1991 | | 3.6 | 59 | 0.88 | 0.37-2.06 | 1982-1991 | | 3 | | 86.9 | 0.91 | | 0.41-1.99 | 1982-1991 | 2.1 | 90.9 | 1.23 | | 0.44-3.45 |  |  |  |  |  |  |  |  |
|  | 1992-2001 | | 4.9 | 114.7 | 1.6 | 0.75-3.37 | 1992-2001 | | 4.4 | | 140 | 1.25 | | 0.66-2.37 | 1992-2001 | 2.2 | 106.2 | 1.29 | | 0.54-3.06 |  |  |  |  |  |  |  |  |
|  | 2002-2011 | | 3 | 85.7 | 1.32 | 0.67-2.59 | 2002-2011 | | 5.2 | | 126.9 | 0.96 | | 0.54-1.71 | 2002-2011 | 2.4 | 101 | 1.03 | | 0.49-2.16 |  |  |  |  |  |  |  |  |
|  | 2012-2015 | | 3.1 | 72.9 | 1 | 1 | 2012-2015 | | 4.4 | | 113.9 | 1 | | 1 | 2012-2015 | 3 | 90 | 1 | | 1 |  |  |  |  |  |  |  |  |
|  | AAPC | | - | - | 1.27% | -1.12- 3.72 | AAPC | | - | | - | 2.43% | | -0.12-5.03 | AAPC | - | - | 0.22% | | -3.23- 2.88 |  |  |  |  |  |  |  |  |
| Physical activity | **Low** | | | | | | **Moderate** | | | | | | | | **High** | | | | |  |  |  |  |  |  |  |  |  |
|  | **Period** | | **N per year** | **ASR** | **HR** | **95% CI** | **Period** | | **N per year** | | **ASR** | **HR** | | **95% CI** | **Period** | **N per year** | **ASR** | HR | | 95% CI |  |  |  |  |  |  |  |  |
|  | 1972-1981 | | 0.8 | 52.7 | 0.55 | 0.11-2.69 | 1972-1981 | | 0.6 | | 46.2 | 0.43 | | 0.17-1.12 | 1972-1981 | 0.2 | 240.7 | 1.98 | | 0.59-6.68 |  |  |  |  |  |  |  |  |
|  | 1982-1991 | | 1.5 | 75.5 | 0.57 | 0.15-2.09 | 1982-1991 | | 2.2 | | 71.1 | 0.62 | | 0.29-1.33 | 1982-1991 | 0.3 | 96.7 | 2.17 | | 0.71-6.64 |  |  |  |  |  |  |  |  |
|  | 1992-2001 | | 1 | 74 | 1.42 | 0.6-3.37 | 1992-2001 | | 5.6 | | 118.5 | 1.07 | | 0.6-1.92 | 1992-2001 | 0.8 | 121.2 | 2.62 | | 0.98-7.03 |  |  |  |  |  |  |  |  |
|  | 2002-2011 | | 0.4 | 65.2 | 1.1 | 0.54-2.25 | 2002-2011 | | 8.6 | | 111.2 | 0.95 | | 0.56-1.62 | 2002-2011 | 1.4 | 100 | 1.94 | | 0.78-4.79 |  |  |  |  |  |  |  |  |
|  | 2012-2015 | | 0.7 | 74.7 | 1 | 1 | 2012-2015 | | 7.7 | | 90.6 | 1 | | 1 | 2012-2015 | 1.7 | 105.7 | 1 | | 1 |  |  |  |  |  |  |  |  |
|  | AAPC | | - | - | 1.71% | -2.26-5.85 | AAPC | | - | | - | 2.4% | | 0-4.87 | AAPC | - | - | 0.65% | | -3.56-2.34 |  |  |  |  |  |  |  |  |
| Other hormonal contraceptives than the IUD | **Non user** | | | | | | **User** | | | | | | | |  |  |  |  |  |  |  |  |  |  |  |  |  |  |
|  | **Period** | | **N per year** | **ASR** | **HR** | **95% CI** | **Period** | | **N per year** | | **ASR** | **HR** | | **95% CI** |  |  |  |  |  |  |  |  |  |  |  |  |  |  |
|  | 1972-1981 | | 2.6 | 68.8 | 0.85 | 0.39-1.82 | 1972-1981 | | 0 | | 0 | 0 | | - |  |  |  |  |  |  |  |  |  |  |  |  |  |  |
|  | 1982-1991 | | 5.3 | 85.6 | 0.99 | 0.5-1.96 | 1982-1991 | | 0.2 | | 21.8 | 0.29 | | 0.05-1.66 |  |  |  |  |  |  |  |  |  |  |  |  |  |  |
|  | 1992-2001 | | 6.8 | 137.6 | 1.41 | 0.78-2.58 | 1992-2001 | | 1.8 | | 107.4 | 1.29 | | 0.59-2.82 |  |  |  |  |  |  |  |  |  |  |  |  |  |  |
|  | 2002-2011 | | 2.5 | 120.4 | 1.27 | 0.66-2.44 | 2002-2011 | | 2.7 | | 67.3 | 0.8 | | 0.41-1.57 |  |  |  |  |  |  |  |  |  |  |  |  |  |  |
|  | 2012-2015 | | 3.7 | 87.2 | 1 | 1 | 2012-2015 | | 3.1 | | 76.6 | 1 | | 1 |  |  |  |  |  |  |  |  |  |  |  |  |  |  |
|  | AAPC | | - | - | 1.06% | -0.83-2.99 | AAPC | | - | | - | 0.95% | | -3.66-5.77 |  |  |  |  |  |  |  |  |  |  |  |  |  |  |

| Risk factor | Value | Person years | Cancers | ASR | Univariate | | Multivariate^[[1]](#footnote-1)^ | |
| --- | --- | --- | --- | --- | --- | --- | --- | --- |
|  |  |  |  |  | HR | 95% CI | HR | 95% CI |
| Smoking | Never | 89629 | 21 | 14.5 | 1.00 |  | 1.00 |  |
|  | Former | 33649 | 13 | 20.5 | 1.45 | 0.72-2.93 | 1.34 | 0.66-2.73 |
|  | Current | 60027 | 18 | 15.7 | 1.24 | 0.66-2.33 | 1.43 | 0.73-2.77 |
| Alcohol | No use | 23423 | 6 | 13.0 | 1.00 |  | 1.00 |  |
|  | Moderate use | 89433 | 33 | 22.3 | 1.12 | 0.45 -2.79 | 0.9 | 0.35-2.30 |
|  | Heavy use | 13416 | 5 | 21.0 | 1.16 | 0.34 -3.97 | 0.9 | 0.25-3.24 |
| Education | Low | 85678 | 20 | 12.1 | 1.00 |  | 1.00 |  |
|  | Intermediate | 57094 | 21 | 20.7 | 1.46 | 0.79-2.69 | 1.43 | 0.77-2.67 |
|  | High | 39499 | 11 | 17.1 | 1.09 | 0.52-2.27 | 1.05 | 0.49-2.25 |
| Parity | Yes | 92743 | 29 | 14.0 | 1.00 |  | 1.00 |  |
|  | No | 61727 | 11 | 11.3 | 0.67 | 0.33 -1.35 | 0.69 | 0.33-1.43 |
| Physical activity | Vigorous | 29919 | 15 | 29.7 | 1.00 |  | 1.00 |  |
|  | Moderately active | 66052 | 18 | 16.2 | 0.55 | 0.27-1.09 | 0.55 | 0.27-1.09 |
|  | Inactive | 41875 | 11 | 13.3 | 0.56 | 0.25-1.24 | 0.60 | 0.27 -1.34 |
| BMI | < 25 kg/m^2^ | 125192 | 42 | 19.1 | 1.00 |  |  |  |
|  | 25–29.9 kg/m^2^ | 33640 | 7 | 11.0 | 0.54 | 0.24-1.21 | 0.53 | 0.24-1.20 |
|  | ≥30 kg/m^2^ | 11993 | 1-4 | 10.3 | 0.60 | 0.19-1.95 | 0.57 | 0.17 -1.89 |
| Use of IUD | Never use | 51947 | 14 | 15.6 | 1.00 |  | 1.00 |  |
|  | Ever use | 4450 | 1-4 | 86.3 | 2.13 | 0.60-7.58 | 1.70 | 0.47-6.23 |
| Hormonal contraceptives other than the IUD | Never use | 72041 | 24 | 15.5 | 1.00 | 1.00 | 1.00 |  |
|  | Ever use | 44493 | 8 | 12.0 | 0.40 | 0.17-0.93 | 0.52 | 0.21-1.30 |

**Supplementary Table 5: Number of EO-BCs, person years, age-standardized rates (ASR) and hazard ratios in women aged 18-39 years by risk factors during 1972-2015**

| Risk factor | Value | Person years | Cancers | ASR | Univariate | | Multivariate^[[2]](#footnote-2)^ | |
| --- | --- | --- | --- | --- | --- | --- | --- | --- |
|  |  |  |  |  | HR | 95% CI | HR | 95% CI |
| Smoking | Never | 140687 | 175 | 121.0 | 1.00 |  | 1.00 |  |
|  | Former | 43503 | 64 | 143.8 | 1.07 | 0.80-1.44 | 1.08 | 0.80-1.45 |
|  | Current | 69533 | 103 | 143.6 | 1.12 | 0.87-1.43 | 1.11 | 0.86-1.43 |
| Alcohol | No use | 33356 | 34 | 98.8 | 1.00 |  | 1.00 |  |
|  | Moderate use | 115148 | 168 | 143.0 | 1.27 | 0.86-1.86 | 1.19 | 0.79-1.78 |
|  | Heavy use | 17779 | 29 | 154.4 | 1.37 | 0.82-2.30 | 1.24 | 0.72-2.14 |
| Education | Low | 111273 | 127 | 112.1 | 1.00 |  | 1.00 |  |
|  | Intermediate | 84928 | 132 | 150.6 | 1.30 | 1.01-1.66 | 1.26 | 0.98-1.63 |
|  | High | 56208 | 81 | 136.7 | 1.23 | 0.93-1.63 | 1.16 | 0.87-1.55 |
| Parity | Yes | 182858 | 233 | 123.7 | 1.00 |  | 1.00 |  |
|  | No | 53953 | 90 | 164.3 | 1.25 | 0.97-1.59 | 1.23 | 0.95-1.58 |
| Physical activity | Vigorous | 33655 | 47 | 136.5 | 1.00 |  | 1.00 |  |
|  | Moderately active | 101051 | 140 | 132.5 | 0.97 | 0.70-1.36 | 1.01 | 0.72-1.40 |
|  | Inactive | 57982 | 86 | 144.6 | 1.12 | 0.78-1.61 | 1.23 | 0.85-1.77 |
| BMI | < 25 kg/m^2^ | 159766 | 240 | 146.9 | 1.00 |  |  |  |
|  | 25–29.9 kg/m^2^ | 64088 | 73 | 108.9 | 0.78 | 0.60-1.01 | 0.78 | 0.60-1.02 |
|  | ≥30 kg/m^2^ | 25758 | 23 | 84.1 | 0.60 | 0.39-0.92 | 0.60 | 0.39-0.92 |
| Use of IUD | Never use | 47093 | 66 | 138.5 | 1.00 |  | 1.00 |  |
|  | Ever use | 9555 | 12 | 122.4 | 0.90 | 0.49-1.68 | 1.01 | 0.54-1.89 |
| Hormonal contraceptives other than the IUD | Never use | 118013 | 163 | 132.8 | 1.00 |  | 1.00 |  |
|  | Ever use | 41049 | 52 | 127.0 | 0.85 | 0.61-1.18 | 0.83 | 0.58-1.20 |

**Supplementary Table 6: Number of EO-BCs, person years, age-standardized rates (ASR) and hazard ratios in women aged 40-49 years by risk factors during 1972-2015**

1. a Adjusted for age, period, alcohol use, BMI, education, use of IUD, use of hormonal contraceptives other than the IUD, smoking, parity and physical activity. Adjustment for variables with structural missing values (alcohol use, parity, physical activity, use of IUD and hormonal contraceptives other than IUD) was partial, with information lacking for certain previously specified cohorts. [↑](#footnote-ref-1)
2. a Adjusted for age, period, alcohol use, BMI, education, use of IUD, use of hormonal contraceptives other than the IUD, smoking, parity and physical activity. Adjustment for variables with structural missing values (alcohol use, parity, physical activity, use of IUD and hormonal contraceptives other than IUD) was partial, with information lacking for certain previously specified cohorts. [↑](#footnote-ref-2)
